# Classification of a new phytoplasmas subgroup 16SrII-W associated with *Crotalaria* witches’ broom diseases in Oman based on multigene sequence analysis

**DOI:** 10.1186/s12866-017-1130-3

**Published:** 2017-11-25

**Authors:** Ali Al-Subhi, Saskia A. Hogenhout, Rashid A. Al-Yahyai, Abdullah M. Al-Sadi

**Affiliations:** 1 Department of Crop Sciences, College of Agricultural and Marine Sciences, Sultan Qaboos University, Al Khod 123, PO Box 34, Seeb, Oman; 2 Department of Crop Genetics, John Innes Centre, Norwich, NR4 7UH UK

**Keywords:** WBD, Phytoplasma phylogeny, Crotalaria

## Abstract

**Background:**

*Crotalaria aegyptiaca*, a low shrub is commonly observed in the sandy soils of wadis desert and is found throughout all regions in Oman. A survey for phytoplasma diseases was conducted. During a survey in a wild area in the northern regions of Oman in 2015, typical symptoms of phytoplasma infection were observed on *C. aegyptiaca* plants. The infected plants showed an excessive proliferation of their shoots and small leaves.

**Results:**

The presence of phytoplasma in the phloem tissue of symptomatic *C. aegyptiaca* leaf samples was confirmed by using Transmission Electron Microscopy (TEM). In addition the extracted DNA from symptomatic *C. aegyptiaca* leaf samples and *Orosius* sp. leafhoppers were tested by PCR using phytoplasma specific primers for the 16S rDNA, *secA*, *tuf* and *imp,* and SAP11 genes. The PCR amplifications from all samples yielded the expected products, but not from asymptomatic plant samples. Sequence similarity and phylogenetic tree analyses of four genes (16S rDNA, *secA*, *tuf* and *imp*) showed that *Crotalaria* witches’ broom phytoplasmas from Oman is placed with the clade of Peanut WB (16SrII) close to Fava bean phyllody (16SrII-C), Cotton phyllody and phytoplasmas (16SrII-F), and *Candidatus* Phytoplasma aurantifolia’ (16SrII-B). However, the *Crotalaria*’s phytoplasma was in a separate sub-clade from all the other phytoplasmas belonging to Peanut WB group. The combination of specific primers for the SAP11 gene of 16SrII-A, −B, and -D subgroup pytoplasmas were tested against *Crotalaria* witches’ broom phytoplasmas and no PCR product was amplified, which suggests that the SAP11 of *Crotalaria* phytoplasma is different from the SAP11 of the other phytoplasmas.

**Conclusion:**

We propose to assign the *Crotalaria* witches’ broom from Oman in a new lineage 16SrII-W subgroup depending on the sequences analysis of 16S rRNA, *secA*, *imp*, *tuf,* and SAP11 genes. To our knowledge, this is the first report of phytoplasmas of the 16SrII group infecting *C. aegyptiaca* worldwide.

**Electronic supplementary material:**

The online version of this article (10.1186/s12866-017-1130-3) contains supplementary material, which is available to authorized users.

## Background

*Crotalaria aegyptiaca* (Benth), a low shrub that reaches about 60 cm high, is commonly observed in the sandy soils of desert wadis [1]. *C. aegyptiaca* is mostly distributed in the Middle East, including Egypt and the Arabian Peninsula. Additionally it spreads throughout all regions in Oman [2, 3]. *C. aegyptiaca* containing pyrrolizidine alkaloids (PAs) [4] is used in traditional medicine [5] and as an antitumor [6]. Sheep and goats do not graze *C. aegyptiaca* because of the Pas’ toxicity, but is grazed by camels and gazelles [3, 5].

**Fig. 1:**
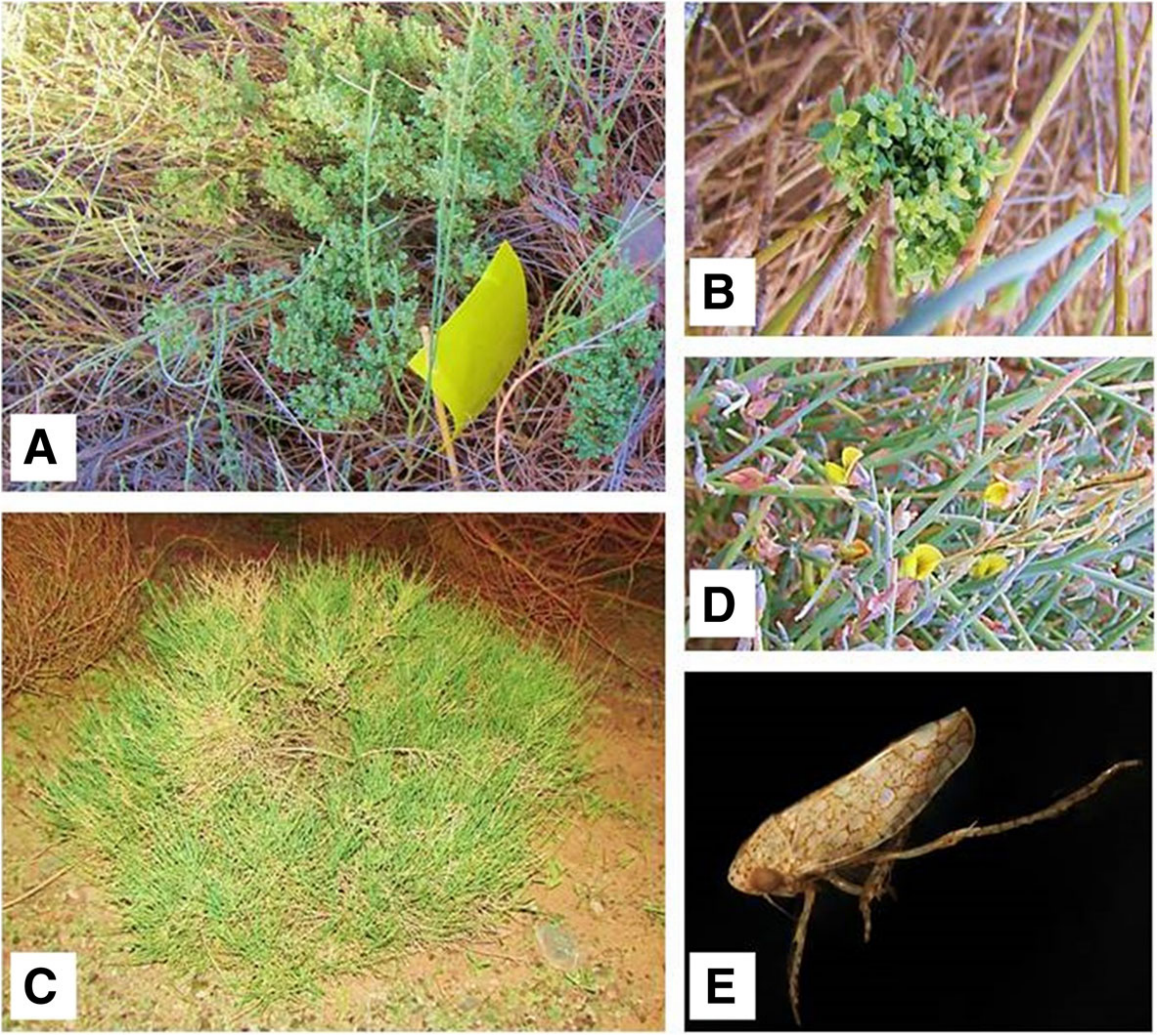
**a** & **b**. is an infected *C. aegyptiaca* plant showing witches’ broom symptoms with an excessive number of little leaves and short shoots; (**c**). & (**d**). is a healthy *C. aegyptiaca* plant; (**e**). is a *Orosius* sp. Leafhopper which was collected from the infected *C. aegyptiaca* sites using yellow sticky traps visible in Fig. 1a

A survey for phytoplasma diseases in wild plants was conducted in the northern regions of Oman in 2015. During this survey, a typical symptom of phytoplasma infection was observed on *C. aegyptiaca* plants in three different locations. The infected plants showed an excessive proliferation of their shoots which is indicative of witch’s broom disease (Fig. 1).

Phytoplasma belonging to the 16SrII-B and -D subgroups have been reported on several economic plants in Oman. Typical symptoms of phytoplasma in acid lime (*Citrus aurantifolia*) showing witches’-broom (WBDL) were first reported in Oman during 1970’s [7]. Zreik et al. [8] identified the 16SrII-B subgroup phytoplasma as a causal agent of WBDL. The 16SrII-B subgroup phytoplasma has only been recorded as a host specific to citrus crops in Oman [8, 9]. Though, more than 20 host plants belonging to different families were found infected with the 16SrII-D subgroup phytoplasma in Oman. Examples include alfalfa [10], sesame (*Sesamum indicum* L.) [11] and chickpea [12].

Phytoplasmas (genus ‘*Candidatus* Phytoplasma’) are intracellular plant pathogenic bacteria in the class Mollicutes [13, 14]. They are transmitted and disseminated into healthy plant phloem from the salivary glands of insect vectors, such as, leafhoppers, planthoppers and psyllids [15–17] and by vegetative propagation [18, 19]. Symptoms associated with phytoplasmas infections include virescence, phyllody, yellowing, stunting, excessive proliferation of shoots, the formation of witches’ brooms, and big bud [20]. Phytoplasmas are obligate parasites so they cannot be cultured on artificial growth media; this makes their identification and characterization difficult. Different methods have been used for the detection of phytoplasma. The first discovery, visual observation and description of phytoplasmas were accomplished with transmission electron microscopy (TEM) in 1967 [21]. TEM was frequently used to provide reliable and accurate methods for diagnosing phytoplasma diseases as well as to get information on the morphology, size, and concentration of phytoplasma bodies in sieve tube elements and insect vectors [22, 23].

The latest developments in the last three decades of molecular-based methods for the detection and identification of phytoplasmas have largely replaced the traditional methods [24]. Furthermore, they have led to a dramatic increase in the understanding of phytoplasmas in the fields of classification, genome sequencing and their interaction with plant hosts and insect vectors. In the early 1990’s, the design of “universal” primers, helped in classification of phytoplasmas [24–26]. For the fine classification of phytoplasmas, to describe subgroups within the 16Sr groups, the 16S rRNA gene is not sufficient due to being highly conserved and is a non-coding gene [27–30]. Many studies have used less-conserved or variable genes as extra molecular markers in conjunction with the 16S rRNA gene for the finer classification of closely related phytoplasma species. The *tuf* gene has been used for 16SrI and 16SrXII subgroup diversity of phytoplasmas [28, 31]. Makarova et al. [32] reported that the *tuf* gene provides a better phytoplasma identification than the 16S rRNA gene. The sequences of *secA* and 23S rRNA genes were used for finer phylogenetic analyses of phytoplasma and their efficient use in phytoplasma disease diagnostics [33]. Three non-homologous protein types including immunodominant membrane protein (Imp), immunodominant membrane protein A (ImpA), and antigenic membrane protein (Amp) were registered as surface membrane protein genes and highly variable genes in the phytoplasmas genome [34–36]. In Iran Siampour et al. [37] used the *imp* gene to characterize and study the phylogenetic trees of several 16SrII-A, −B, and -C subgroups of phytoplasma strains that cause the disease in various host plants in Iran, East Asia, Africa, and Australia. The phytoplasmas induce symptoms by secretion of SAP11 effector protein. SAP11 modify the plant-gene activity and has a role in genetic regulator of the changed phenotype produced [38, 39]. The SAP11 gene (effector proteins) contains eukaryotic nuclear localization signals (NLS) that localize in plant cell nuclei and interfere with plant TCP (TEOSINTE BRANCHED1, CYCLOIDEA, PROLIFERATING CELL FACTORS 1 and 2), which are conserved gene among plant species [38, 40]. The SAP11 transgenic *Arabidopsis thaliana* plants show crinkled leaves and produce multiple stems; moreover, these symptom, down regulate Jasmonic acid (JA) synthesis and modulated phosphate (Pi) homeostasis [38, 41].

Phytoplasmas are disseminated into healthy plant phloem from the salivary glands of phloem-feeding and sap sucking insect vectors, belonging to hemipteran order including the families *Cicadellidea* (leafhoppers), *Fulgoridea* (planthoppers) and *Psylloidea* (psyllids) insect while feeding [16]. The insect vectors spread phytoplasma diseases [42], so a successful phytoplasma management is to reduce and control the insect vectors [43]. Two leafhopper species, *Austroagallia avicula* and *Empoasca* sp., were registered as putative vectors of alfalfa witches’ broom phytoplasma in Oman [44].

The objective of this study is to diagnose and detect the causal agent and potential insect vector species for the phytoplasma-like symptoms (Witches’ Broom) of *C. aegyptiaca* in Oman by using electron microscopy and molecular approaches to define a detailed classification of the causal agent of the *C. aegyptiaca* disease. This detailed classification was achieved by comparing multiple gene regions including 16S rDNA, *secA*, *tuf*, *imp* and SAP11 genes.

## Methods

### Samples collection

*C. aegyptiaca* infected samples showing phytoplasma-like symptoms (witches’ broom) were collected from three sites in Oman, in Al-Seeb (N: 23.587794, E: 58.317110) area from the Muscat governorate and two locations are from Al-Dakhilia governorates which were Samail (N: 23.082359, E: 57.819496) and Izki (N: 22.911003, E: 57.741790). Healthy and infected samples showing typical witches’ broom symptoms of *C. aegyptiaca* were collected from the sampled locations and stored at −80 **°**C until used. Sampling was done after getting the necessary permissions from plant owners Leafhoppers were collected from the three sites using yellow sticky traps that were placed near the infected plants for 5 days in order to investigate the putative insect vectors of the pathogen using molecular detection techniques.

### Transmission electron microscopy (TEM)

Fresh midribs of symptomatic *C. aegyptiaca* leaf samples were placed in karnovesky’s fixative (2% Gluteraldehyde and 4% paraformaldehyde containing 1 M cacodylate buffer) overnight at 4 **°**C. After fixation the samples were washed in two 10-min cycles of a 1 M cacodylate buffer (pH from 7.2 to 7.4) then left overnight in a 1 M cacodylate buffer. In secondary fixation, the samples were then placed in 1% osmium tetraoxide (OsO_4_) (prepared in distilled water, pH 7.2) for 1 h on a rotary shaker, followed by washing in distilled water for two 20-min cycles. The samples were then dehydrated in increasing gradients of acetone (25%, 75% and 95%) for 15 min, followed by 3 cycles of absolute acetone in the following order, 2 cycles for 30 min each and third cycle for 1 h to ensure the complete removal of water. Specimens were then infiltrated with an acetone/resin (1:1) mixture at room temperature on rotators overnight. After that the samples were then transferred to acetone/resin (1:3) mixture overnight on a rotator. Further 2 cycles of fresh 100% araldite resin were carried out for 1 h each. Each piece of mid-rib was placed in a standard mold block (capsules) with labels, fresh resin was added in the capsules, and they were incubated at 60 °C overnight for polymerization. Sections of 0.5 μm thicknesses were cut with glass knives using an Ultra Microtome and stained with a 1% toluidine blue dye and examined by through an optical microscope to determine the area for ultra-thin sectioning. Ultra-thin sections of 70 nm thicknesses were cut using an Ultra Microtome fitted with a diamond knife. The ultra-thin sections were placed in holders called GRIDS (300 mesh) then stained with aqueous uranyl acetate for 30 min and lead citrate for 25 min. Finally, the stained ultra-thin sections were examined using a Jeol Jem-2100S transmission electron microscope at the Electron Microscopy Unit in Sultan Qaboos University.

### DNA extraction and PCR amplification

Four symptomatic and four asymptomatic *C. aegyptiaca* samples as well *Orosius sp.* leafhoppers (Table 1) from the three sites of this study in Oman were included in the DNA extraction. Total DNA was extracted from 0.1 g of plant samples and 5 to 10 specimens from each leafhoppers species using the Doyle and Doyle [45] method with some modifications. The sample tissue powder was immediately added in nucleic acid extraction buffer (100 mM Tris- HCl at pH 8.0, 1% cetyltrimethylammonium bromide [CTAB)], 2% PVP-10, 1.4 M NaCl, 20 mM EDTA, and 0.1% 2-mercaptoethanol), then 1% sodium dodecyl sulfate (SDS) was added. The sample was incubated for 30 min at 65 °C.Total DNA was extracted with an equal volume of phenol-chloroform-isoamyl alcohol (25:24:1). The total DNA was precipitated with 0.6 volumes of isopropanol and 0.3 M sodium acetate. Then the DNA pellet was washed twice with 70% ethanol, dried, and re-suspended in 100 μL of TE buffer (10 mM Tris-HCl, 1 mM EDTA) and the DNA was stored at −20 **°**C until use. Polymerase Chain Reactions (PCR) for five gene sequences including 16S rDNA, *secA*, *tuf*, *imp,* and SAP11 genes were performed for the detection, diagnostic, and phylogenetic studies of the phytoplasma that is associated with the *C. aegyptiaca* witches’ broom disease. Phytoplasma DNA extracted from lime witches’ broom (WBDL) (Table 1) and alfalfa witches’ broom disease (AlWD) (Table 1) were used as positive control, whereas the negative control samples had been DNA extracted from asymptomatic plants and sterile water. Primer pairs P1/P7 [26, 46] and R16F2n/R16R2 [47] were used, as a direct and nested PCR respectively, to amplify the entire 16Sr RNA gene region at annealing temperatures of 55 **°**C and 60 **°**C respectively. The 16SrII group phytoplasmas specific primers sequences (Table 2) were used to amplify *secA*, *tuf*, *imp,* and SAP11 genes sequences. The *secA* gene was partially amplified using the primer pair SecA-II-F1/SecA-II- R1 as the direct PCR and primer pair SecA-II-F1/SecA-II-R4 as the semi-nested PCR at an annealing temperature of 53 **°**C. A portion of the *tuf* genes were amplified with primer pairs TUF-II-F1/TUF-II-R1 and TUF-II-F2/TUF-II-R1, as the direct and the semi-nested PCR respectively, at an annealing temperature of 53 **°**C. PCR was conducted to amplify the full length of the *imp* and SAP11 genes sequences of the phytoplasma that is associated with the *C. aegyptiaca* witches’ broom disease in Oman at an annealing temperature of 53 **°**C. Primer pairs IMP-II-F1/IMP-II-R1 and IMP-II-F2/IMP-II-R1, as the direct and the semi-nested PCR reactions respectively, were used to amplify the *imp* gene. The specific primers for the SAP11 genes of 16SrII-D subgroup and 16SrII-B subgroup pytoplasmas were utilized in direct and nested PCR including the SAP11-IID-F1/SAP11-IID-R1 and the SAP11-IID-F2/SAP11-IID-R2 primers pairs of 16SrII-D subgroup phytoplasma and excluding the SAP11-WBDL-F1/SAP11-WBDL-R1 (direct PCR) and the SAP11-WBDL-F2/SAP11-WBDL-R2 (direct PCR) for the 16SrII-B subgroup phytoplasma. PCR reactions were performed in ‘Ready-To-Go’ PCR beads (Pharmacia Biotech, Sweden) with 25 μl reaction volumes, which contain 50 ng of genomic DNA, 0.5 μl of each primer (10 pmoles); the reaction volumes were adjusted with sterile deionized water. The PCR was performed for 40 cycles using the following parameters: denaturation at 94 **°**C for 45 s (2 min for the first cycle), annealing for 1 min at X **°**C (X = annealing temperature specified for each set of primers), and primer extension at 72 **°**C for 1.5 min with a final extension cycle for 10 min at 72 **°**C. The resulting PCR products were visualized by electrophoresis in a 1.4% agarose gel, stained with ethidium bromide, placed under a UV transilluminator, and photographed. The PCR products were purified and sequenced at Macrogen Company (South Korea).

**Table 1:** List of phytoplasma isolates of the 16Sr-II group used in this study with their source and geographical origins, and corresponding 16S rDNA, *secA*, *imp,* and *tuf* gene accession numbers

Isolate number	Phytoplasma strain	Isolate	Source	Acronym	Location/governorate	Accession numbers*			
						16S rDNA	*imp*	*secA*	*tuf*
1	*Crotalaria* witches’ broom	SQU-Sa1	*C. aegyptiaca*	CrWBDL	Samail/Al-Dakhilia	KY872734	KY872727	KY872719	KY872723
2	*Crotalaria* witches’ broom	SQU-Sa2	*C. aegyptiaca*	CrWBDL	Samail/Al-Dakhilia	KY872735	KY872728	KY872720	KY872724
3	*Crotalaria* witches’ broom	SQU-Iz1	*C. aegyptiaca*	CrWBDL	Izki/Al-Dakhilia	KY872736	KY872729	KY872721	KY872725
4	*Crotalaria* witches’ broom	SQU-Se1	*C. aegyptiaca*	CrWBDL	Al-Seeb/Muscat	KY872737	KY872730	KY872722	KY872726
5	*Crotalaria* witches’ broom	SQU-Sa3	*Orosius* sp.	OrWBDL	Samail/Al-Dakhilia	KY872738	KY872731	–	–
6	*Crotalaria* witches’ broom	SQU-Iz2	*Orosius* sp.	OrWBDL	Izki/Al-Dakhilia	KY872739	KY872732	–	–
7	*Crotalaria* witches’ broom	SQU-Se2	*Orosius* sp.	OrWBDL	Al-Seeb/Muscat	KY872740	KY872733	–	–
10	Lime witches’ broom	SQU-LW1	Lime	WBDL	Al-Seeb/Muscat	KX358574	KX358610	KX358586	KX358598
11	Alfalfa witches’ broom	SQU-Al1	Alfalfa	AlWB	Al-Najed/Dhofar	KX358564	KX358600	KX358576	KX358588

^*^The deposited accession numbers in GenBank can be accessed by blasting the accession numbers through the following link

https://blast.ncbi.nlm.nih.gov/Blast.cgi?PROGRAM=blastn&PAGE_TYPE=BlastSearch&LINK_LOC=blasthome

**Table 2:** Primers used for PCR amplification and sequencing of the 16S rRNA, *secA, imp, tuf*, and SAP11 genes of the phytoplasma

Primer Sets	Location	PCR product size	Reaction	Sequence (5′ to 3′)
SecA-II-F1/SecA-II-R1	secA gene	2141 bp	Direct PCR	AAAGATGAAGATTTTCCTAAAGA/TCCATATCATTTATATGACGTTGA
SecA-II-F1/SecA-II-R4	secA gene	1511 bp	Semi-Nested PCR	AAAGATGAAGATTTTCCTAAAGA/ACAAAAAATTTAGTATAACCAGGATC
IMP-II-F1/IMP-II-R1	imp gene	786 bp	Direct PCR	GTTATAATTGAAGGCGATATTG/ATAGAGGAGAAGAAAAAGTTTCT
IMP-II-F2/IMP-II-R1	imp gene	717 bp	Semi-Nested PCR	GATCATATTTGGTTTATAGGAG/ATAGAGGAGAAGAAAAAGTTTCT
TUF-II-F1/TUF-II-R1	tuf gene	1490 bp	Direct PCR	GCTTTTGTTCCTTTAGCAGAA/AGACTATACACTAGTCTTCTT
TUF-II-F2/TUF-II-R1	tuf gene	1094 bp	Semi-Nested PCR	CGCAAAGATATTAAAACTTTAG/AGACTATACACTAGTCTTCTT
SAP11-IID-F1/SAP11-IID-R1	SAP11 gene	820 bp	Direct PCR	CGGCAAAATAAAAGTTCAAATCA/AATCGAAACCAACCAACTTATAG
SAP11-IID-F2/SAP11-IID-R2	SAP11 gene	465 bp	Nested PCR	TTCTCAATTAAACGAACTCTACG/AAAAAGACCCTTCAGAAAGGGTC
SAP11-WBDL-F1/SAP11-WBDL-R1	SAP11 gene	1050 bp	Direct PCR	CTTCAGCCACAAATAGAATCTTT/CAAATACAAATCGCTGCATAAA
SAP11-WBDL-F2/SAP11-WBDL-R2	SAP11 gene	550 bp	Nested PCR	TTCCTTTTATGAAATCACCTCAG/GCGCATATTATTAAACTCCTTT

### Sequence analysis and construction of phylogenetic trees

The DNA sequences of both strands, forward and reverse, of each sample were edited, assembled, and aligned using BioEdit 7.0.4.1 [48]; the sequences were adjusted manually where it was necessary. The resulting sequences of the phytoplasma 16S rDNA, *secA*, *tuf,* and *imp* genes were compared to phytoplasma species available in the National Center for Biotechnology (NCBI) GenBank database (http://ncbi.nlm.nih.gov/BLAST) by BLAST searches to identify homologous sequences. The DNA sequences were deposited in GenBank (NCBI, Bethesda, MD, USA) under the accession numbers in Table 1.

Sequence data of the phytoplasma 16S rDNA, *secA*, *tuf,* and *imp* genes were obtained from GenBank to study the genetic relationships of the collected *C. aegyptiaca* phytoplasma samples to known phytoplasma groups. Sequences were aligned using CLUSTAL W [49] then checked and confirmed manually. Sequences from our study were aligned with 56 16S rDNA, 29 *secA*, 14 *imp*, and 38 *tuf* gene sequences of reference strains from GenBank (Additional file 1: Table S1 and Table S2). A partition-homogeneity test (PHT) in the PAUP* 4.0b10 [50] package was implemented to test whether data for the 16S rDNA, *secA*, *imp,* and *tuf* genes regions could be combined in a single tree. The combined tree has phytoplasma sequences of the four genes and is available in the GenBank (Additional file 1: Table S2). The phylogenetic analysis of the 16S rDNA, *secA*, *imp,* and *tuf* genes as well as the tree of combined genes were carried out with MEGA 6 software [51]. The neighbour-joining method was used to construct the phylogenetic trees with 1000 replications for bootstrap analysis and a Kimura-2-parameter model [52]. The DNA sequences of *Bacillus subtilis* (AB042061), *B. subtilis* (D10279) and *B. subtilis* strain 168 (GCA_000789275) were used as the out-groups taxa of the trees of 16S rDNA, *secA*, and *tuf* genes respectively.

### Virtual RFLP analysis

Computer-simulated RFLP analysis was performed using iPhyClassifier (https://plantpathology.ba.ars.usda.gov/cgi-bin/resource/iphyclassifier.cgi) tools [53] for the DNA sequence of 16S rRNA gene (1242 bp) phytoplasma from *C. aegyptiaca* samples and *Orosius* sp. leafhopper samples compared with all 21 strains of16SrII group phytoplasmas (Additional file 1: Table S1). The pDRAW32 software (http://www.acaclone.com) were used to perform virtual RFLP plotting of 16S rRNA gene sequence from *C. aegyptiaca* samples and *Orosius* sp. leafhopper samples and 16SrII-M subgroup phytoplasmas.

## Results

### Symptomatology and leafhopper identification

During the survey on phytoplasma diseases in wild plants of Oman in 2015, *C. aegyptiaca* showed symptoms indicative of a phytoplasma disease from three sites. Symptoms included the significant proliferation of shoots, reduced stem height, and an increased number of leaves compared to healthy plants and at the same time witches’ broom symptoms were observed with the progress of the disease symptoms (Fig. 1a to c). All of the yellow sticky traps which were placed near the infected *C. aegyptiaca* in the three sites, consistently collected brown leafhopper specimens that were 3.8–4.2 mm in length size, these were identified as *Orosius* sp. (Fig. 1e).

### Transmission electron microscopy (TEM)

**Fig. 2:**
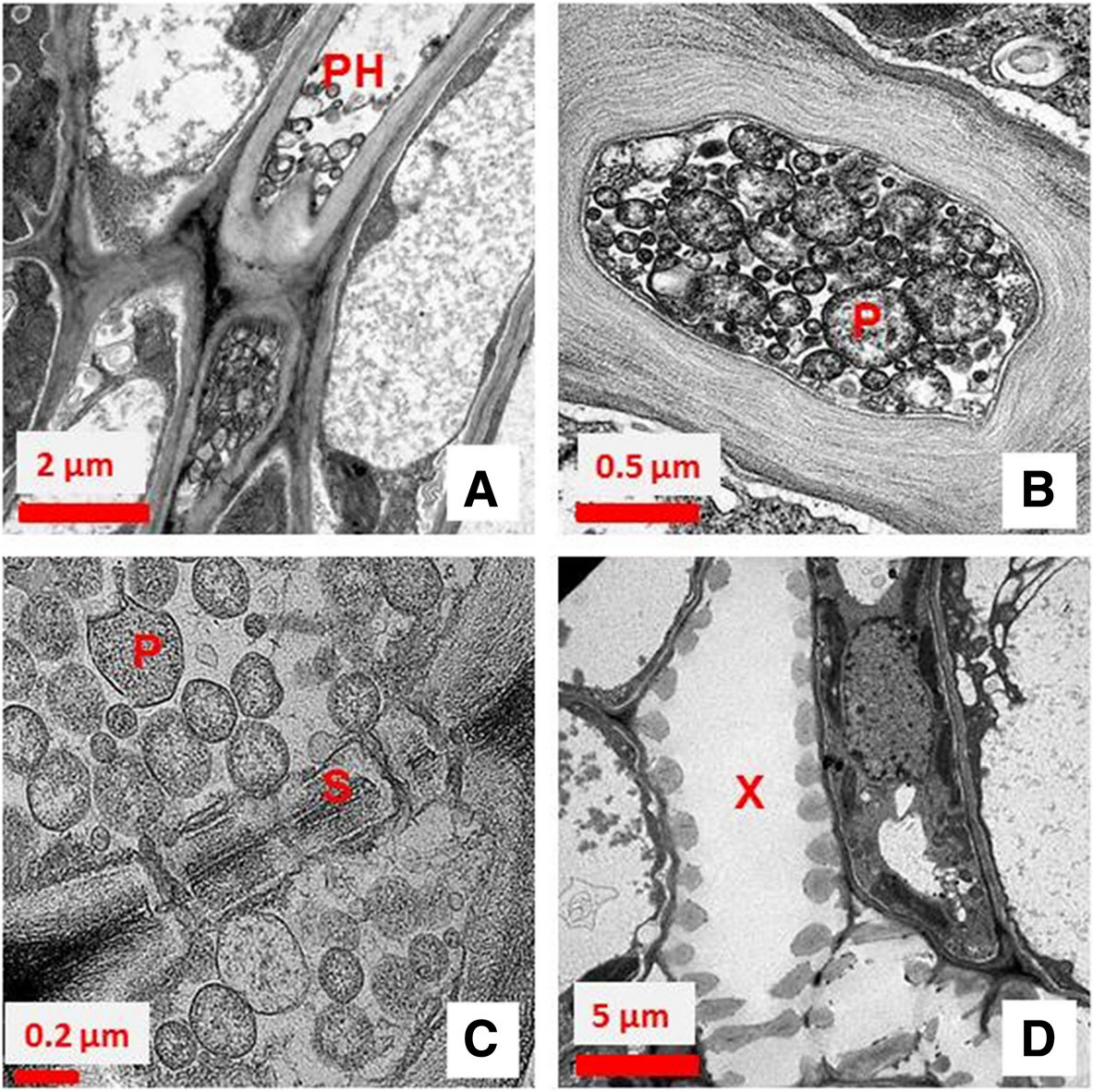
Transmission electron micrograph of phytoplasma cells within the phloem of minor veins of infected *C. aegyptiaca* witches’ broom leaf. **a** elongationsection showing phytoplasma within the sieve tube (phloem) (Ph); **b** A cross section showing a high concentration of phytoplasma bodies in sieve tubes P = phytoplasma cell; **c** elongation section showing the sieve plate (S) of sieve tubes and that phytoplasma cells have moved through sieve plate pores; **d** Xylem cell (X) was free from phytoplasma cells

Examination of ultra-thin cross and elongation sections of *C. aegyptiaca* leaf midrib, from witches’ broom infected plants, showed numerous phytoplasma bodies in the sieve tubes (Fig. 2a & b). The observed phytoplasma cells were spherical to ovoid measuring 200–600 nm in diameter, enclosed by a single unit outer membrane (Fig. 2c). The distribution of phytoplasma cells was irregular in the infected phloem. Some phloem elements, mainly concentrated along sieve plates, showed phytoplasma bodies in large abundance and were almost clogged (Fig. 2c). No phytoplasma bodies were found in the xylem tissues of infected leaves (Fig. 2d). As a result, the TEM images confirmed that phytoplasma is the causal agent of the *C. aegyptiaca* witches’ broom disease in Oman.

### PCR amplification and sequence analyses to detect phytoplasma: 16S rDNA

**Fig. 3:**
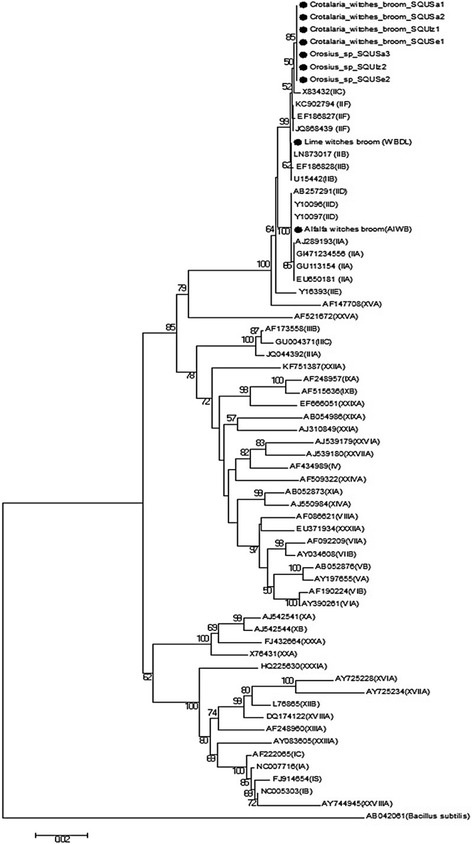
Phylogenetic tree of the 16S rRNA gene sequences from the four *C. aegyptiaca* phytoplasma and three *Orosius* sp. Leafhopper isolates plus the Lime witches broom sample (WBDL) and the Alfalfa witches’ broom (AlWB) from Oman (with circular black shape). The tree also includes 56 phytoplasma strains from previously published sequences, shown in GenBank accession numbers, and 16S groups are indicated in brackets (Additional file 1: Table S1); it was rooted using *Bacillus subtilis* (AB042061). The phylogenetic tree was constructed by the neighbour-joining method and Kimura’s two-parameter model, and is in the units of the number of base substitutions per site. The bootstrap values are expressed as percentages of 1000 replications

Symptomatic and asymptomatic *C. aegyptiaca* samples and *Orosius* sp. leafhopper samples were utilized for the detection of phytoplasma by amplifying the 16S rRNA gene. The alfalfa witches’ broom (AlWB) and lime witches’ broom samples (WBDL) were used as the positive control and a water sample as the negative control. The direct and nested PCR assay were conducted with P1/P7 and R16F2n/R16R2 primer pairs and yielded the expected amplification fragment length of about 1.8 kb and 1.2 kb fragment respectively (data not shown) from all of the symptomatic *C. aegyptiaca* and entire *Orosius* sp. leafhopper samples tested. The asymptomatic plants and water samples tested tested negative. PCR tests confirmed the association of phytoplasma diseases with *C. aegyptiaca* plants and *Orosius* sp. leafhoppers in Oman. The 16S rRNA gene sequence analyses of 4 *C. aegyptiaca* phytoplasmas and three *Orosius* sp. leafhopper isolates confirmed the PCR results. The sequences were deposited in the National Center for Biotechnology (NCBI) database under the accession numbers listed in Table 1. The sequence homology of *C. aegyptiaca* isolates and *Orosius* sp. leafhopper isolates of 16S rRNA gene phytoplasma were 100% identical to each other and >99% and 99% similar with that of the Lime (WBDL) and alfalfa (AlWB) phytoplasmas respectively, which served as controls. The BLAST searches at NCBI of the 16S rRNA gene from *C. aegyptiaca* phytoplasma and *Orosius* sp. leafhopper isolates showed that these isolates have 99% sequence similarity with Cotton phyllody phytoplasma strain CoP (Accession. No. JQ868439) described as the 16SrII-F subgroup, and also with *Candidatus* Phytoplasma aurantifolia strain 37oman (Accession. No. LN873017) and the Iranian apple phytoplasma (Accession. No. KC902794) belonging to the 16SrII-B subgroup ‘*Candidatus* Phytoplasma aurantifolia’. Therefore, the *C. aegyptiaca* phytoplasma is a member of the 16SrII group phytoplasmas and is closely related to ‘*Candidatus* Phytoplasma aurantifolia’. As shown in Fig. 3, the Neighbor-Joining phylogenetic tree, derived from partial 16S rDNA (1151 bp) sequences data, placed all seven phytoplasmas isolates of *Crotalaria* witches’ broom phytoplasmas from *C. aegyptiaca* and *Orosius* sp. leafhoppers from Oman in one separate sub-clade within the 16SrII group (Peanut WB) clade with 85% bootstrap support. They were found to be closely related to the Fava bean phyllody (Accession. No. X83432), from 16SrII-C subgroup phytoplasmas, and they were further away from lime witches’ broom ‘Ca. P. aurantifolia’ (WBDL) (Fig. 3). The virtual RFLP patterns analysis results using the *i*PhyClassifier software and the pDRAW32 software for the 16S rDNA R16F2n/R16R2 fragment sequence phytoplasma from *C. aegyptiaca* samples and *Orosius sp*. leafhopper samples clearly distinguished our phytoplasma from the 21 strains of16SrII group phytoplasmas (Additional file 2: Figure S1, Additional file 3: Figure S2). The virtual RFLP analysis gave identical results with the 16S rDNA phylogenetic tree finding.

### DNA sequences analysis:- *secA*, *imp*, *tuf,* and SAP11 genes

The *secA*, *tuf,* and *imp* genes sequence with amplicon size ~1370 bp, ~996 bp, and 516 bp respectively of *Crotalaria* witches’ broom phytoplasmas from *C. aegyptiaca* and just *imp* gene sequence from *Orosius* sp. leafhoppers phytoplamsa from Oman were submitted to the GenBank NCBI database under the accession numbers in Table 1. Sequences of *secA*, *tuf, and imp* genes from *C. aegyptiaca* and *Orosius* sp. leafhoppers from Oman samples were 100% identical to each other. BLAST search of the *secA* gene at NCBI showed that *Crotalaria* witches’ broom phytoplasmas from Oman had a 98% similarity with the sequence of primula blue yellow phytoplasma (Accession. No. KJ462018) and a 97% similarity with witches’ broom disease of lime phytoplasma (‘*Candidatus* Phytoplasma aurantifolia’) sequence (Accession. No. KJ462017). Moreover the sequence of the *tuf* gene showed that *Crotalaria* witches’ broom phytoplasmas had a 98% similarity with a phytoplasma associated with primula blue yellow disease (Accession. No. JQ824229) and had a 97% similarity with witches’ broom disease of lime phytoplasma (Accession. No. JQ824276). Nonetheless, the sequence identity of the imp gene of *Crotalaria* witches’ broom phytoplasmas from Oman showed only a 90% similarity with primula blue yellow disease (Accession. No. JQ745272) and witches’ broom disease of lime (Accession. No. JQ745278) phytoplasmas. The combination of specific primers for the SAP11 gene of 16SrII-A, −B, and -D subgroup phytoplasmas were tested against *Crotalaria* witches’ broom phytoplasmas and no PCR product was amplified. However, the positive control samples gave the expected PCR amplicon size, therefore, there is homology of the SAP11 DNA sequence of *Crotalaria* witches’ broom phytoplasma unlike the SAP11 DNA sequence of 16SrII-A, −B, and -D subgroup pytoplasmas. So, the sequences analysis of *secA*, *imp*, *tuf,* and SAP11 genes confirmed the 16S rRNA gene result to classify *Crotalaria* witches’ broom phytoplasma in the 16SrII-W subgroup.

### Phylogenetic analysis

**Fig. 4:**
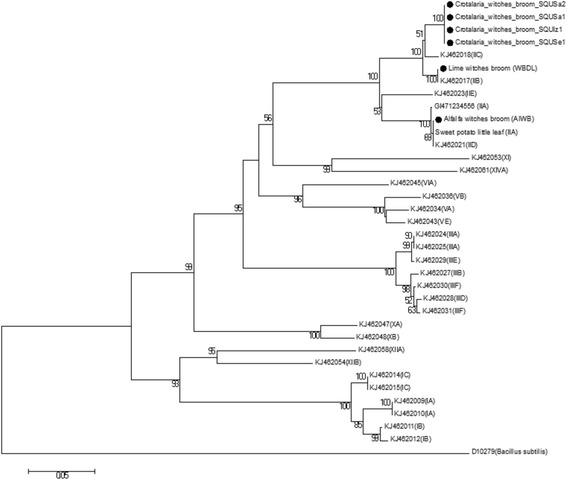
Phylogenetic tree of the *secA* gene sequences from the four *C. aegyptiaca* phytoplasma isolates plus the Lime witches broom sample (WBDL) and the Alfalfa witches’ broom (AlWB) from Oman (with circular black shape). The tree also includes 29 phytoplasma strains from previously published sequences, shown in GenBank accession numbers, and 16S groups that are indicated in brackets (Additional file 1: Table S2); it was rooted using *Bacillus subtilis* (D10279). The phylogenetic tree was constructed by the neighbour-joining method and Kimura’s two-parameter model, and is in the units of the number of base substitutions per site. The bootstrap values are expressed as percentages of 1000 replications

**Fig. 5:**
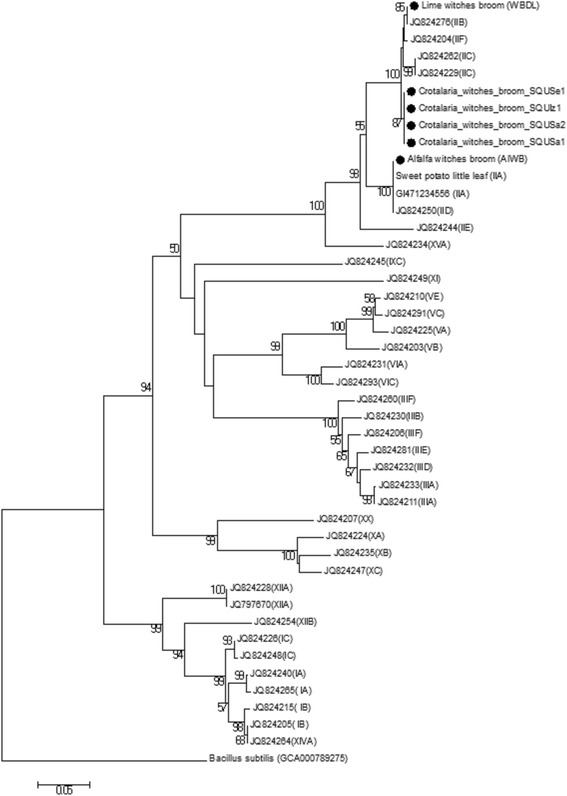
Phylogenetic tree of the *tuf* gene sequences from the four *C. aegyptiaca* phytoplasma isolates plus the Lime witches broom sample (WBDL) and the Alfalfa witches’ broom (AlWB) from Oman (with circular black shape). The tree also includes 38 phytoplasma strains from previously published sequences, shown in GenBank accession numbers, and 16S groups that are indicated in brackets (Additional file 1: Table S2); it was rooted using *Bacillus subtilis* strain 168 (GCA_000789275). The phylogenetic tree was constructed by the neighbour-joining method and Kimura’s two-parameter model, and is in the units of the number of base substitutions per site. The bootstrap values are expressed as percentages of 1000 replications

**Fig. 6:**
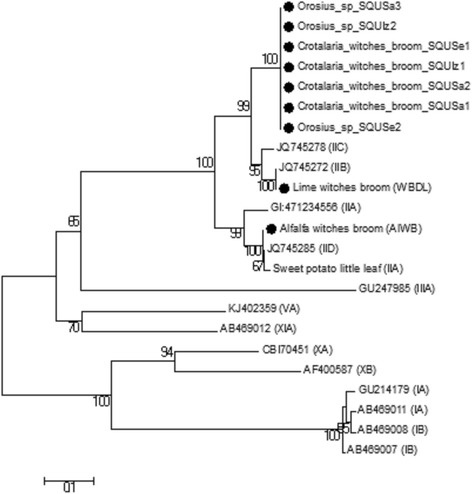
Phylogenetic tree of the *imp* gene sequences from the four *C. aegyptiaca* phytoplasma and three *Orosius* sp. Leafhopper isolates plus the Lime witches broom sample (WBDL) and the Alfalfa witches’ broom (AlWB) from Oman (with circular black shape). The tree also includes 14 phytoplasma strains from previously published sequences, shown in GenBank accession numbers, and 16S groups that are indicated in brackets (Additional file 3: Table S2). The phylogenetic tree was constructed by the neighbour-joining method and Kimura’s two-parameter model, and is in the units of the number of base substitutions per site. The bootstrap values are expressed as percentages of 1000 replications

**Fig. 7:**
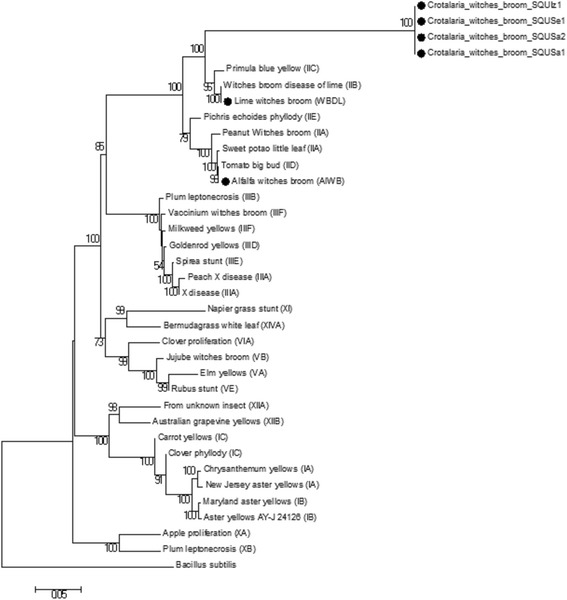
Phylogenetic tree of the 16S rRNA, *secA, tuf,* and *imp* genes sequences from the four *C. aegyptiaca* phytoplasma isolate plus the Lime witches broom sample (WBDL) and the Alfalfa witches’ broom (AlWB) from Oman (with circular black shape). The tree also includes 29 16S rDNA, *secA, and tuf* phytoplasma sequences and 14 *imp* phytoplasmas sequences retrieved from the GenBank, shown in phytoplasma disease names, and 16S groups that are indicated in brackets (Additional file 1: Table S2). The phylogenetic tree was constructed by the neighbour-joining method and Kimura’s two-parameter model, and is in the units of the number of base substitutions per site. The bootstrap values are expressed as percentages of 1000 replications

The DNA sequences of SecA, IMP, and TUF genetic markers from this study were used to construct the phylogenetic trees based on partial sequences of the *secA* and tuf genes, 536 bp (Fig. 4) and 385 bp (Fig. 5) nucleotides respectively as well as complete sequences of *imp* gene 516 bp nucleotides (Fig. 6). Moreover, the phylogenetic tree of the combined dataset was built; this included the 16S rRNA, secA, tuf, and imp gene sequences (Fig. 7). The phylogenetic trees of secA, tuf, and imp gene sequences and combined tree separated the phytoplasma 16Sr group’s lineage which is similar with that inferred by the 16S rRNA gene-based phylogeny in this study (Fig. 3). The 16SrII phytoplasma group was comprised of two sub clades on all four phylogenetic trees (Figs 4 to 7). In these analyses, all isolates of *Crotalaria* witches’ broom phytoplasmas from Oman were 100% identical and clustered in a subclade in section 16SrII- B, −C, and –F subgroup pytoplasmas, but they were clearly separated into an individual subgroup, supported with a very strong bootstrap analysis.

## Discussion

*C. aegyptiaca* showed symptoms typical of phytoplasma infection. The symptoms of *C. aegyptiaca* witches’ broom disease are similar to those of lime witches’ broom disease (WBDL) [54] in Oman, associated with ‘*Candidatus* Phytoplasma aurantifolia’ (16SrII-B subgroup) [8]. TEM showed numerous phytoplasma bodies in the sieve tubes of *C. aegyptiaca*. TEM is frequently used to provide a reliable and accurate method for diagnosing phytoplasma diseases in sieve tube elements and insect vectors [22, 23]. González et al. [55] used TEM to observe maize bushy stunt phytoplasma within its plant host and insect vector. TEM also was applied to detect the phytoplasma associated with sunflower phyllody in India [56] and for elm yellows group phytoplasma that is associated with camellia in China [57].

Our findings showed that *C. aegyptiaca* phytoplasma is a member of the 16SrII group phytoplasmas and is closely related to ‘*Candidatus* Phytoplasma aurantifolia’. Analysis based on the 16S rDNA placed all seven phytoplasmas isolates of *Crotalaria* witches’ broom phytoplasmas from *C. aegyptiaca* and *Orosius* sp. leafhoppers from Oman in one separate sub-clade within the 16SrII group (Peanut WB) clade with 85% bootstrap support. The four phytoplasma groups, including 16SrII-B & -D, 16SrVI, 16SrIX, and 16SrXXIX, infect wild and economically important plant species in Oman [8, 58–60]. The virtual RFLP analysis gave identical results with the 16S rDNA phylogenetic tree finding. The above results showed that *Crotalaria* witches’ broom phytoplasmas from Oman placed with the clade of Peanut WB (16SrII) including Fava bean phyllody (16SrII-C), Cotton phyllody and phytoplasmas (16SrII-F), and ‘*Candidatus* Phytoplasma aurantifolia’ (16SrII-B) (Fig. 3) [8, 29, 61]. To our knowledge, this is the first report of phytoplasmas of the 16SrII group infecting *C. aegyptiaca* worldwide. Previously, many studies reported *Crotalaria* sp. plants infected with phytoplasma diseases. A phytoplasma belonging to 16SrII-A subgroup has been reported to be associated with disease in *Crotalaria szemaoensis* and *Crotalaria zanzibarica* plants in China [62]. A 16SrII-A subgroup phytoplasma was also observed on *Crotalaria* spp. (sunn hemp) in Myanmar [63]. *Crotalaria juncea* (sunn hemp) plants were found to be infected with the 16SrIX group phytoplasma in Brazil [64, 65]. Above all, the phytoplasma associated with *Crotalaria* witches’ broom in Oman seemed to be distinguishable from all the other phytoplasmas belonging to Peanut WB group (16SrII). The 16SrII group has 21 subgroups including 16SrII-A, −B, −C, −D, −E, and –F [66] therefore, we propose to assign the *Crotalaria* witches’ broom from Oman in a new lineage 16SrII-W subgroup. The *Orosius sp.* was registered as phytoplasmas putative vectors in many studies. Pilkington et al. [67] reported *Orosius argentatus* as a vector of the Australian lucerne yellows phytoplasma. In addition, *Orosius albicinctus* was the vector insect of the sesame phyllody disease which the 16SrIX-C and 16SrII-D subgroup phytoplasmas were the causal agent in Turkey, Iran, and India [68, 69]. Thus, *Orosius* sp. leafhoppers from Oman could be responsible for the transmission of inoculum of *Crotalaria* witches’ broom phytoplasmas from infected to healthy *C. aegyptiaca* plants.

Proposing a new ‘Candidatus Phytoplasma’ species can be done if the 16S rRNA gene sequence has less than 97.5% similarity according to International Phytoplasma Working Group (IPWG) [70]. The phytoplasma that shares more than 97.5% of the 16S rRNA gene sequence similarity and has unique ecological and biological properties such as a specific plant host or insect vector could be designated as a separate candidate species [20, 71]. The finer classification and description of the biology and ecology of phytoplasmas that are closely related but distinct strains cannot be easily resolved by the highly conserved 16S rRNA gene alone [30]. Therefore, less conserved markers including *secA*, *imp*, *tuf*, ribosomal protein (rp), secY, and SAP11 genes, have been utilized for finer classification of closely related phytoplamsas within or between the existing16S group or subgroup [27, 29, 31–33, 35]. Findings from our study showed that the sequences analysis of *secA*, *imp*, *tuf,* and SAP11 genes confirmed the 16S rRNA gene result to classify *Crotalaria* witches’ broom phytoplasma in the 16SrII-W subgroup. In adition, results of the phylogenetic trees on *secA*, *tuf,* and *imp* gene sequences and the tree of the four combined genes revealed that *Crotalaria* witches’ broom phytoplasma (*C. aegyptiaca* isolates and *Orosius* sp. Leafhoppers isolates) from Oman is a new phytoplasma, having closer relationships to the phytoplasmas associated with primula blue yellow disease (16SrII-C) than the *Candidatus* Phytoplasma aurantifolia’ (16SrII-B) [8, 29, 32, 33, 72].

## Conclusions

On the basis of disease symptoms and the molecular analysis of 16S rRNA, *secA*, *tuf,* and *imp* genes, phytoplasma isolates from the *C. aegyptiaca* plant and *Orosius* sp. leafhopper isolates in Oman were found to be associated with the *Crotalaria* witches’ broom disease and is a member of the 16SrII group phytoplasma. No PCR amplification came from the SAP11 primer sets, which shows the SAP11 gene of *Crotalaria* witches’ broom phytoplasma has different DNA sequences than 16SrII-B and -D subgroup phytoplasmas. These results support the conclusion that *Crotalaria* witches’ broom phytoplasmas from Oman are closely related to phytoplasmas belonging to the 16SrII-C and -B group, but that it represents a distinct subgroup of phytoplasma. Therefore, we suggested classifying it as the new subgroup 16SrII-W. The results from this study are supported by the usage of multiple genetic markers, which is useful in the fine differentiation and analysis of closely related phytoplasma strain lineages, and might be extremely important for phytoplasma disease epidemiological studies or for disease control and quarantine guidelines. The *Orosius* sp. leafhopper is a putative vector for *Crotalaria* witches’ broom phytoplasma, but the specific transmission tests need to be conducted to confirm that it is in fact a vector. A field survey will be helpful to define the economic crops and alternative plant hosts which are also visited by this leafhopper. Such studies will provide methods toward disease control that could prevent the spread of *Crotalaria* witches’ broom phytoplasma to economic crops.

## Additional files

Additional file 1: Table S1. 16S rDNA sequences of different phytoplasma strains obtained from GenBank used for phylogenetic analysis. **Table S2**. Phytoplasma 16S rRNA, tuf, secA, and imp genes sequences used for phylogenetic analysis, obtained from GenBank. (DOCX 23 kb)

Additional file 2: Figure S1. Virtual RFLP patterns by the *i*PhyClassifier software of the 16S rRNA gene phytoplasmas from *C. aegyptiaca* samples, *Orosius* sp. leafhopper samples and all 21 16SrII group strains using *Alu*I, *Bga*I, *Bst*VI, *Eco*RI, *Hae*III, *Hha*I, *Hin*fI, *Hpa*II, *Mse*I, *Sau*3AI, *Ssp*I and *Taq*I restriction endonuclease enzymes. (PPTX 184 kb)

Additional file 3: Figure S2. Virtual RFLP comparative analysis with different restriction enzymes of 16S DNA sequences of phytoplasma from *C. aegyptiaca* samples and *Orosius* sp. leafhopper samples and 16SrII-M subgroup phytoplasmas using the pDRAW32 software. (PPTX 200 kb)

## Abbreviations

AlWB: Almond witches’ broom ‘Ca. P. phoenicium’
AP: Apple proliferation ‘Ca. *P. mali*’
AshY: Ash yellows ‘Ca. *P. fraxini*’
AUSGY: Australian grapevine yellows ‘Ca. *P. australiense*’
AYWB: *Aster yellows witches’ broom (AYWB)*
BGWL: Bermudagrass white leaf ‘Ca. P. cynodontis’
CaWB: Cassia witches’ broom (CaWB) ‘Ca. P. omanense’
COAH10: Mexican potato purple top phytoplasma strain COAH10
CP: Clover proliferation ‘Ca. *P. trifolii*’
CPh: Clover phyllody (CPh)
CTAB: Cetyltrimethylammonium bromide
CYE: Clover yellow edge
EDTA: Ethylenediaminetetraacetic acid
ErWB: Erigeron witches’ broom
ESFY: European stone fruit yellows ‘Ca. P. prunorum’
EY: Elm yellows ‘Ca. *P. ulmi*’
HibWB: Hibiscus witches’ broom ‘Ca. *P. brasiliense*’
*imp*: Immunodominant membrane protein
JWB-G1: Jujube witches’ broom ‘Ca. *P. ziziphi*’
LufWB: Loofah witches’ broom
LYDM: Lethal yellow disease Mozambique ‘Ca. P. palmicola’
MaPV: Malaysian p. virescence (MaPV) ‘Ca. P. malaysianum’
MC: Strawberry multiplier disease
MPV: Mexican periwinkle virescence
OY-M: Onion yellows mild strain (OY-M)
PB: Pecan bunch
PCR: polymerase chair reaction
PinP: Pinus phytoplasma ‘Ca. *P. pini*’
PnWB: Peanut witches’ broom
PnWB: Peanut WB phytoplasma
PPWB: Pigeon pea witches’ broom
PX11CT1: Peach X-disease ‘Ca. *P. pruni*’
RYD: Rice yellow dwarf ‘Ca. *P. oryzae*’
SAP11: Stress associated protein 11
*secA*: preprotein translocase subunit SecA
SoyST1c1: Soybean stunt isolate SoyST1c1 ‘Ca. P. costaricanum’
SQU: Sultan Qaboos University
TEM: Transmission electron microscopy
TexPp: Texas Phoenix palm phytoplasma
tuf: Elongation factor Tu
