# Supplementary material for: Classification of a new phytoplasmas subgroup 16SrII-W associated with Crotalaria witches’ broom diseases in Oman based on multigene sequence analysis
Source: BMC Microbiol. 2017 Nov 25;17:221. doi: 10.1186/s12866-017-1130-3 (PMC5702145; doi:10.1186/s12866-017-1130-3)
Supplement: Supplementary file 1 — 16S rDNA sequences of different phytoplasma strains obtained from GenBank used for phylogenetic analysis. Table S2. Phytoplasma 16S rRNA, tuf, secA, and imp genes sequences used for phylogenetic analysis, obtained from GenBank. (DOCX 23 kb) [file 12866_2017_1130_MOESM1_ESM.docx]

**Supplementary table 1:** 16S rDNA sequences of different phytoplasma strains obtained from GenBank used for phylogenetic analysis.

| Accession Numbers | 16S rDNA groups | Acronym | Phytoplasma strains |
| --- | --- | --- | --- |
| NC_007716 | I-A | AYWB | *Aster yellows witches’ broom (AYWB)* |
| NC_005303 | I-B | OY-M | Onion yellows mild strain (OY-M) |
| AF222065 | I-C | CPh | Clover phyllody (CPh) |
| GI:471234556 | II-A | PnWB | Peanut witches’ broom |
| L33765 | II-A | PnWB | Peanut WB phytoplasma |
| U15442 | II-B |  | Ca. Phytoplasma aurantifolia |
| X83432 | II-C |  | Faba bean phyllody phytoplasma |
| Y10097 | II-D |  | Ca. Phytoplasma australasiae |
| Y16393 | II-E |  | Picris echiode sphyllody phytoplasma |
| EU099556 | II-F |  | Cactus WBphytoplasma – YN11 |
| EU099568 | II-G |  | Cactus WB phytoplasma– YN23 |
| EU099569 | II-H |  | Cactus WB phytoplasma– YN24 |
| EU099551 | II-I |  | Cactus WBphytoplasma– YN06 |
| EU099552 | II-J |  | Cactus WBphytoplasma– YN07 |
| EU099572 | II-K |  | Cactus WBphytoplasma– YN28 |
| EU099546 | II-L |  | Cactus WBphytoplasma– YN01 |
| HG792252 | II-M |  | Tephrosia purpurea WB phytoplasma |
| JF781309 | II-N |  | Bunchy top symptom phytoplasma-IIN-LT |
| EF647744 | II-O |  | Tabebuia pentaphylla phytoplasma |
| DQ286948 | II-P |  | Cuban papaya phytoplasma |
| JF781310 | II-Q |  | Papaya bunchy top phytoplasma-BTSpHav02-IIA |
| DQ535900 | II-R |  | Echinopsis sp. yellow patch phytoplasma |
| FJ357164 | II-S |  | Amaranthus hypochondriacus phytoplasma-52A |
| EU125185 | II-T |  | Tomatillo WB phytoplasma |
| KP057205 | II-U |  | Papaya little leaf phytoplasma– DZ01 |
| JQ044392 | III-A | PX11CT1 | Peach X-disease ‘Ca. P. pruni’ |
| AF173558 | III-B | CYE | Clover yellow edge |
| GU004371 | III-C | PB | Pecan bunch |
| FJ914654 | I-S | COAH10 | Mexican potato purple top phytoplasma strain COAH10 |
| AF434989 | IV | TexPp | Texas Phoenix palm phytoplasma |
| AF248957 | IX-A | PPWB | Pigeon pea witches’ broom |
| AF515636 | IX-B | AlWB | Almond witches’ broom ‘Ca. P. phoenicium’ |
| AY197655 | V-A | EY | Elm yellows ‘Ca. P. ulmi’ |
| AB052876 | V-B | JWB-G1 | Jujube witches’ broom ‘Ca. P. ziziphi’ |
| AY390261 | VI-A | CP | Clover proliferation ‘Ca. P. trifolii’ |
| AF190224 | VI-B | MC | Strawberry multiplier disease |
| AF092209 | VII-A | AshY | Ash yellows ‘Ca. P. fraxini’ |
| AY034608 | VII-B | ErWB | Erigeron witches’ broom |
| AF086621 | VIII-A | LufWB | Loofah witches’ broom |
| AJ542541 | X-A | AP | Apple proliferation ‘Ca. P. mali’ |
| AJ542544 | X-B | ESFY | European stone fruit yellows ‘Ca. P. prunorum’ |
| AB052873 | XI-A | RYD | Rice yellow dwarf ‘Ca. P. oryzae’ |
| L76865 | XII-B | AUSGY | Australian grapevine yellows ‘Ca. P. australiense’ |
| AF248960 | XIII-A | MPV | Mexican periwinkle virescence |
| AJ550984 | XIV-A | BGWL | Bermudagrass white leaf ‘Ca. P. cynodontis’ |
| AB054986 | XIX-A |  | Chestnut witches’ broom ‘Ca. P. castaneae’ |
| AF147708 | XV-A | HibWB | Hibiscus witches’ broom ‘Ca. P. brasiliense’ |
| AY725228 | XVI-A |  | Sugarcane yellow leaf syndrome ‘Ca. P. graminis’ |
| AY725234 | XVII-A |  | Papaya bunchy top ‘Ca. P. caricae’ |
| DQ174122 | XVIII-A |  | American potato purple top wilt ‘Ca. P. americanum’ |
| X76431 | XX-A |  | Rhamnus witches’ broom ‘Ca. P. rhamni’ |
| AJ310849 | XXI-A | PinP | Pinus phytoplasma ‘Ca. P. pini’ |
| KF751387 | XXII-A | LYDM | Lethal yellow disease Mozambique ‘Ca. P. palmicola’ |
| AY083605 | XXIII-A |  | Buckland valley grapevine yellows |
| AF509322 | XXIV-A |  | Sorghum bunchy shoot |
| EF666051 | XXIX-A | CaWB | Cassia witches’ broom (CaWB) ‘Ca. P. omanense’ |
| AF521672 | XXV-A |  | Weeping tea witches broom |
| AJ539179 | XXVI-A |  | Sugarcane phytoplasma strain D3T1 |
| AJ539180 | XXVII-A |  | Sugarcane phytoplasma strain D3T2 |
| AY744945 | XXVIII-A |  | Derbid phytoplasma |
| FJ432664 | XXX-A |  | Salt cedar witches’ broom ‘Ca. P. tamaricis’ |
| HQ225630 | XXXI-A | SoyST1c1 | Soybean stunt isolate SoyST1c1 ‘Ca. P. costaricanum’ |
| EU371934 | XXXII-A | MaPV | Malaysian p. virescence (MaPV) ‘Ca. P. malaysianum’ |

**Supplementary table 2**: Phytoplasma 16S rRNA, *tuf, secA,* and *imp* genes sequences used for phylogenetic analysis, obtained from GenBank

| **Phytoplasma strain Phytoplasma** | **16Sr Group** | **Abbreviation** | **Accession number** | | | |
| --- | --- | --- | --- | --- | --- | --- |
|  |  |  | **16S rRNA gene *** | ***tuf* gene** | ***secA* gene** | ***imp* gene** |
| Chrysanthemum yellows | I-A | CHRYM | AY265214 | JQ824240 | KJ462009 | GU214179 |
| New Jersey aster yellows | I-A | NJ-AY | HM590622 | JQ824265 | KJ462010 | AB469011 |
| Maryland aster yellows | I-B | AY-1 | AF322644 | JQ824205 | KJ462011 | AB469008 |
| Aster yellows AY-J 24126 | I-B | AY-J | HM590616 | JQ824215 | KJ462012 | AB469007 |
| Clover phyllody | I-C | KVE | AY265217 | JQ824248 | KJ462014 |  |
| Carrot yellows | I-C | CA | HM448473 | JQ824226 | KJ462015 |  |
| Peanut witches’ broom | II-A | PnWB | GI:471234556 | GI:471234556 | GI:471234556 | GI:471234556 |
| Witches’ broom disease of lime | II-B | WBDL | EF186828 | JQ824276 | KJ462017 | JQ745272 |
| Faba bean phyllody | II-C | FBP |  | JQ824262 |  |  |
| Primula blue yellow | II-C | PrBY | JQ868438 | JQ824229 | KJ462018 | JQ745278 |
| Tomato big bud | II-D | TBB-KG | Y08173 | JQ824250 | KJ462021 | JQ745285 |
| Sweet potato little leaf | II-A | SPLL | AJ289193 | Draft Genome | Draft Genome | Draft Genome |
| Pichris echoides phyllody | II-E | PEP | Y16393 | JQ824244 | KJ462023 |  |
| Peach X disease | III-A | CX | HQ589202 | JQ824211 | KJ462024 | GU247985 |
| X disease | III-A | BF | HQ589203 | JQ824233 | KJ462025 |  |
| Plum leptonecrosis | ﻿III-B | LNI | JQ868444 | JQ824230 | KJ462027 |  |
| Goldenrod yellows | III-D | GRI | GU004372 | JQ824232 | KJ462028 |  |
| Spirea stunt | III-E | SPI | HQ589206 | JQ824281 | KJ462029 |  |
| Vaccinium witches’ broom | III-F | VAC | HQ589201 | JQ824260 | KJ462030 |  |
| Milkweed yellows | III-F | MW1 | HQ589200 | JQ824206 | KJ402031 |  |
| Elm yellows | V-A | EY1 | AY197655 | JQ824225 | KJ462034 | KJ402359 |
| Jujube witches’ broom | V-B | JWB | AY197661 | JQ824203 | KJ462036 |  |
| Rubus stunt | V-E | RuS | AY197648 | JQ824210 | KJ462043 |  |
| Clover proliferation | VI-A | CP1 | AY390261 | JQ824231 | KJ462045 |  |
| Catharanthus phyllody | VI-C | CPS |  | JQ824293 |  |  |
| Apple proliferation | X-A | AT | CU469464 | JQ824224 | KJ462047 | CBI70451 |
| Plum leptonecrosis | X-B | LNp | JQ868450 | JQ824235 | KJ462048 | AF400587 |
| Napier grass stunt | XI | NGS-BS | JQ868440 | JQ824249 | KJ462053 | AB469012 |
| From unknown insect | XII-A | BA | JQ868436 | JQ824228 | KJ462058 |  |
| Australian grapevine yellows | XII-B | AGY | NC_010544 | JQ824254 | KJ462054 |  |
| Bermudagrass white leaf ‘Ca. P. cynodontis’ | XIV-A |  | AJ550984 | JQ824264 | KJ462061 |  |
| Stolbur ‘Ca. P. solani’ | XII-A | STOL11 |  | JQ797670 |  |  |
| Suriname virescence - | XV-A | SUV |  | JQ824234 |  |  |
| Rhamnus cathartica stunt - | XX | RhCa |  | JQ824207 |  |  |
| Cotton phyllody - | II-F | CoP |  | JQ824204 |  |  |
| ”Flavescence dorée” - FD-C Serbia 86/09 | V-C | FD-C |  | JQ824291 |  |  |
| Naxos yellows -NAXOS | IX-C | NAXOS |  | JQ824245 |  |  |
| Pear decline - PD | X-C | PD |  | JQ824247 |  |  |

***The Gene bank accession strains of the 16S rRNA gene is used in the combined phylogenetic tree only.**
