# Supplementary figures and images for: Classification of a new phytoplasmas subgroup 16SrII-W associated with Crotalaria witches’ broom diseases in Oman based on multigene sequence analysis
Source: BMC Microbiol. 2017 Nov 25;17:221. doi: 10.1186/s12866-017-1130-3 (PMC5702145; doi:10.1186/s12866-017-1130-3)

## Slide 1
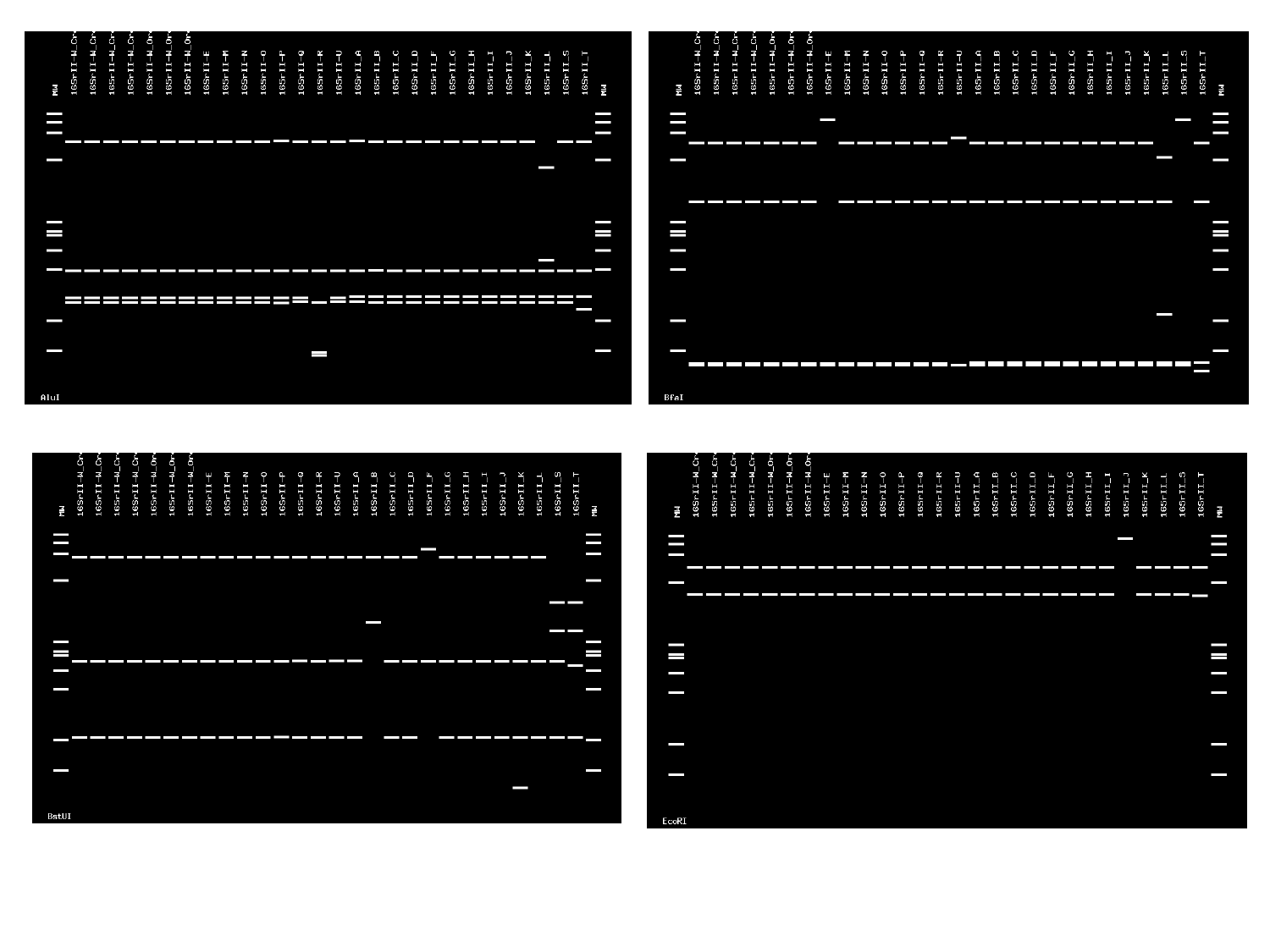

## Slide 2
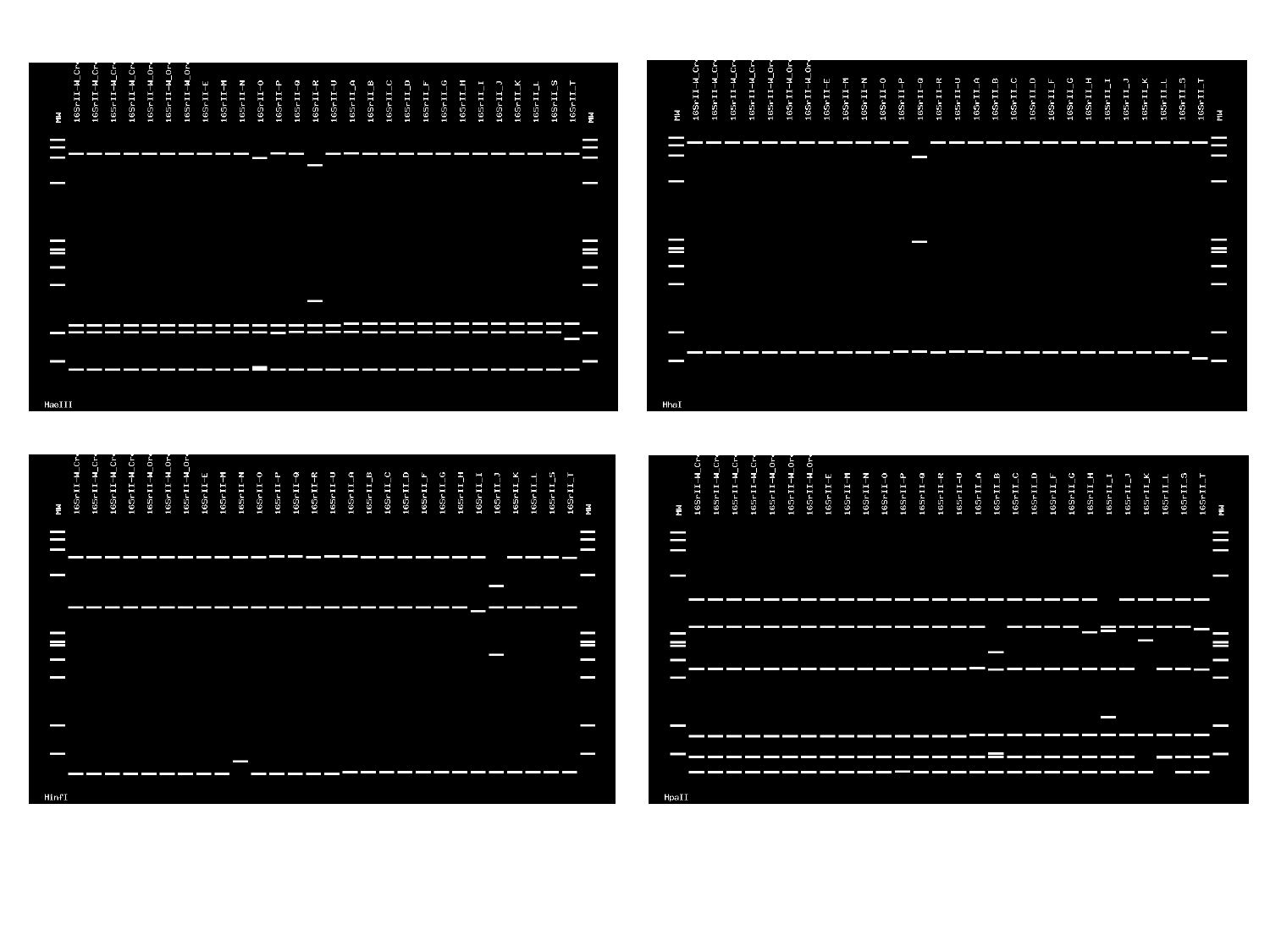

## Slide 3
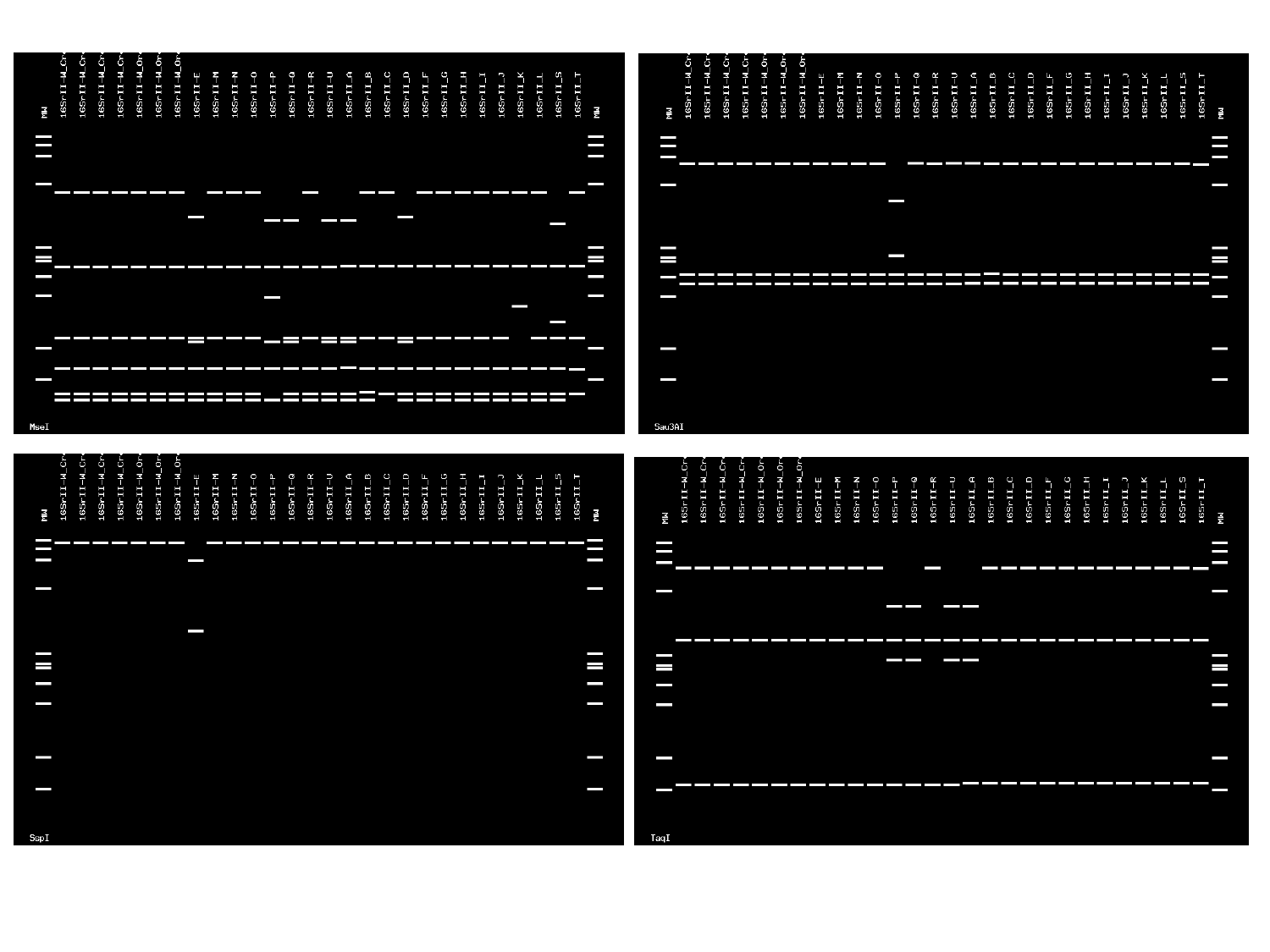

Supplement: Supplementary file 2 — Virtual RFLP patterns by the iPhyClassifier software of the 16S rRNA gene phytoplasmas from C. aegyptiaca samples, Orosius sp. leafhopper samples and all 21 16SrII group strains using AluI, BgaI, BstVI, EcoRI, HaeIII, HhaI, HinfI, HpaII, MseI, Sau3AI, SspI and TaqI restriction endonuclease enzymes. (PPTX 184 kb) [file 12866_2017_1130_MOESM2_ESM.pptx]

## Slide 1
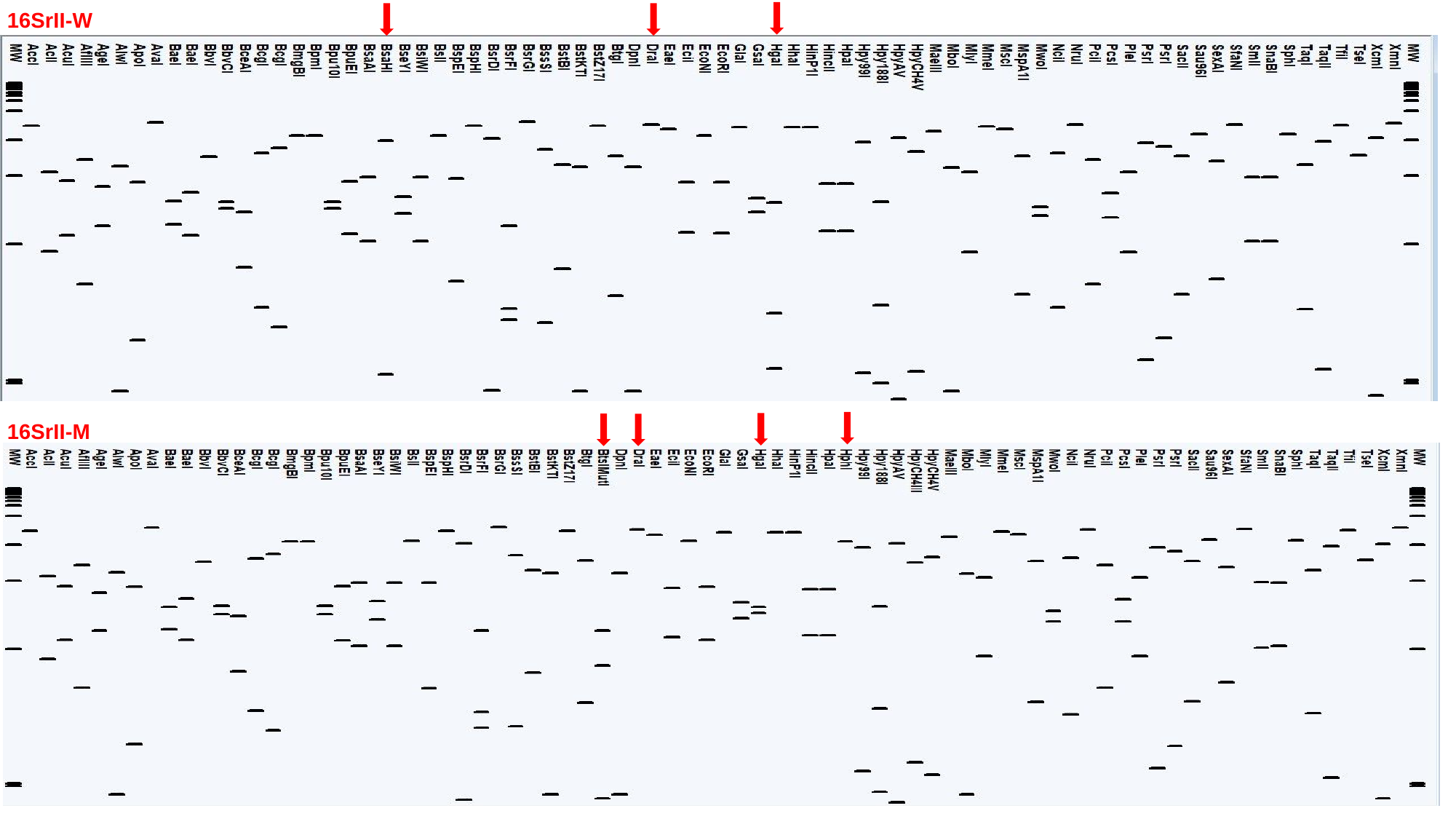

16SrII-W
16SrII-M

Supplement: Supplementary file 3 — Virtual RFLP comparative analysis with different restriction enzymes of 16S DNA sequences of phytoplasma from C. aegyptiaca samples and Orosius sp. leafhopper samples and 16SrII-M subgroup phytoplasmas using the pDRAW32 software. (PPTX 200 kb) [file 12866_2017_1130_MOESM3_ESM.pptx]
